# Supplementary material for: Comparative transcriptomics identifies candidate genes involved in the evolutionary transition from dehiscent to indehiscent fruits in Lepidium (Brassicaceae)
Source: BMC Plant Biol. 2022 Jul 14;22:340. doi: 10.1186/s12870-022-03631-8 (PMC9281134; doi:10.1186/s12870-022-03631-8)
Supplement: Supplementary file 1 — Additional file 1: Supplementary Data 1. Perl script for the detection of chimeric transcripts in the trinity assembly of the Lepidium transcriptomes. [file 12870_2022_3631_MOESM1_ESM.docx]

**Supplementary Data 1:** Perl script for the detection of chimeric transcripts in the trinity assembly of the Lepidium transcriptomes.

#! /usr/bin/perl

use strict;

use warnings;

main();

sub main {

# define variables

my $blast;

my $transcriptome;

my $orthologs;

my $output_folder;

# read commandline arguments

foreach (my $i=0; $i<@ARGV; $i++){

if ($ARGV[$i] eq "-b"){

$blast = $ARGV[$i+1];

}

if ($ARGV[$i] eq "-t"){

$transcriptome = $ARGV[$i+1];

}

if ($ARGV[$i] eq "-r"){

$orthologs = $ARGV[$i+1];

}

if ($ARGV[$i] eq "-o"){

$output_folder = $ARGV[$i+1];

}

}

if ((! defined $blast) || (! defined $output_folder)){

#print "Missing arguments\nCall program like this: perl detect_chimers.pl -o <path-to-output-folder>\n";

die();

}

identify_chimers($blast, $transcriptome, $orthologs, $output_folder);

}

sub identify_chimers {

my ($blast, $transcriptome, $orthologs, $output_folder) = @_;

my $isoform;

my %isoform_lengths;

my $subject;

my %subjects;

my %descs;

my %lengths;

my %allengths;

my %qstarts;

my %qends;

my %chimers;

my $coverage1;

my $coverage2;

my %coverages;

my %chimer_subjects;

my $orth_gene;

my %seen;

my $is_chimer = 0;

open(TABLE,">$output_folder/chimer_table.txt");

print TABLE "ID\tisoform\tlength\tchimer\tsubject 1\tsubject 2\tlength 1\talign length 1\talign start 1\talign end 1\tlength 2\talign length 2\talign start 2\talign end 2\tcoverage 1\tcoverage 2\n";

open(BLAST, $blast);

while (<BLAST>){

chomp($_);

if ($_ =~ /^(TRINITY_DN\d+_c\d+_g\d+_i\d+)\s+(\d+)\s+(AT\d+G\d+)\.(\d+)\s+(.+)\s+(\d+)\s+(\d+)\s[0-9\.]+\s+(\d+)\s+(\d+)\s+\d+\s+\d+\s+\d+/){

$isoform = $1;

$isoform_lengths{$isoform} = $2;

$subject = "$3.$4";

$descs{$subject} = $5;

$lengths{$subject} = $6;

$allengths{$isoform}{$subject} = $7;

$qstarts{$isoform}{$subject} = $8;

$qends{$isoform}{$subject} = $9;

if (defined($subjects{$isoform})){

my @subjs = split(/,/,$subjects{$isoform});

foreach my $subj (@subjs){

if ($subj =~ /(AT\d+G\d+)\.\d+/){

$orth_gene = $1;

}

# if another isoform of this subject was not analysed before

if ((!(defined($seen{$isoform}))) || (!($seen{$isoform} =~ /$orth_gene/))){

# if alignment of subjects does not overlap

if ((($qstarts{$isoform}{$subject} < $qstarts{$isoform}{$subj}) & ($qends{$isoform}{$subject} < $qstarts{$isoform}{$subj})) | (($qstarts{$isoform}{$subject} > $qends{$isoform}{$subj}) & ($qstarts{$isoform}{$subject} > $qends{$isoform}{$subj}))){

$is_chimer = split_chimers($isoform, $isoform_lengths{$isoform}, $subject, $subj, $transcriptome, $orthologs, $output_folder);

if ($is_chimer){

print TABLE "$isoform\t$isoform_lengths{$isoform}\t";

print TABLE "chimer of $subject and $subj\t$descs{$subject}\t$descs{$subj}\t";

print TABLE "$lengths{$subject}\t$allengths{$isoform}{$subject}\t";

print TABLE "$qstarts{$isoform}{$subject}\t$qends{$isoform}{$subject}\t";

print TABLE "$lengths{$subj}\t$allengths{$isoform}{$subj}\t";

print TABLE "$qstarts{$isoform}{$subj}\t$qends{$isoform}{$subj}\n";

$is_chimer = 0;

}

if (defined($seen{$isoform})){

$seen{$isoform} .= "$subject, $subj, ";

}

else {

$seen{$isoform} = "$subject, $subj, ";

}

}

}

}

$subjects{$isoform} .= ",$subject";

}

else {

$subjects{$isoform} = "$subject";

}

}

}

close(BLAST);

close(TABLE);

}

sub split_chimers {

my ($isoform, $iso_length, $subject1, $subject2, $transcriptome, $orthologs, $output_folder) = @_;

open(LOG,">>$output_folder/split.log");

print LOG "Trying to split $isoform (lengths = $iso_length) into orthologs of $subject1 and $subject2\n";

my $is_chimer = 0;

my $read = 0;

my $query = "";

my $seq = "";

# read assembly - get isoform

open(TRANSCR, $transcriptome);

open(QUERY, ">$output_folder/query.fasta");

while(<TRANSCR>){

chomp($_);

if ($_ =~ /$isoform\s+/){

print QUERY "$_\n";

$read = 1;

}

elsif ($_ =~ /$isoform$/){

print QUERY "$_\n";

$read = 1;

}

elsif (($read) && ($_ =~ /^>/)){

print QUERY "$query\n";

$read = 0;

}

elsif ($read){

$query .= $_;

}

}

close(QUERY);

close(TRANSCR);

$read = 0;

$seq = "";

# read Arabidopsis transcripts - get orthologs

open(ORTH, $orthologs);

open(SUBJ, ">$output_folder/subjects.fasta");

while(<ORTH>){

chomp($_);

if (($_ =~ /$subject1\s+/) || ($_ =~ /$subject2\s+/)) {

if ($read){

print SUBJ "$seq\n";

}

print SUBJ "$_\n";

$read = 1;

$seq = "";

}

elsif (($read) && ($_ =~ /^>/)){

print SUBJ "$seq\n";

$read = 0;

}

elsif ($read){

$seq .= $_;

}

}

close(SUBJ);

close(ORTH);

# conduct blast search

my $blastsearch = "blastn -query $output_folder/query.fasta -subject $output_folder/subjects.fasta -gapextend 2 -penalty -1 -outfmt \"6 qseqid qlen sseqid slen qstart qend sstart send\" >$output_folder/$isoform\_$subject1\_$subject2.blast";

system $blastsearch;

# determine split position

my $subj;

my %len;

my $qstart;

my %qend;

my %sstart;

my %send;

my %min_qstart;

open(BLAST2, "$output_folder/$isoform\_$subject1\_$subject2.blast");

print LOG "BLAST result\n";

while(<BLAST2>){

chomp($_);

if ($_ =~ /^$isoform\s+\d+\s+([A-Za-z0-9\.]+)\s+(\d+)\s+(\d+)\s+(\d+)\s+(\d+)\s+(\d+)/){

$subj = $1;

$len{$subj} = $2;

$qstart = $3;

$qend{$subj}{$qstart} = $4;

$sstart{$subj}{$qstart} = $5;

$send{$subj}{$qstart} = $6;

if (defined $min_qstart{$subj}){

if ($qstart < $min_qstart{$subj}){

$min_qstart{$subj} = $qstart;

}

}

else {

$min_qstart{$subj} = $qstart;

}

}

print LOG "$_\n";

}

close(BLAST2);

# BLAST hit found for both putative orthologs

if ((defined($min_qstart{$subject1})) && (defined($min_qstart{$subject2}))){

my $first_transcript;

my $last_transcript;

if ($min_qstart{$subject1} < $min_qstart{$subject2}){

$first_transcript = $subject1;

$last_transcript = $subject2;

}

else {

$first_transcript = $subject2;

$last_transcript = $subject1;

}

print LOG "first transcript: $first_transcript\n";

print LOG "last transcript: $last_transcript\n";

my $max_qend1 = 1;

my $max_qstart1;

foreach my $qs (keys %{$qend{$first_transcript}}){

if ($qend{$first_transcript}{$qs} > $max_qend1){

$max_qend1 = $qend{$first_transcript}{$qs};

$max_qstart1 = $qs;

}

}

my $min_qstart2 = $iso_length;

foreach my $qs (keys %{$qend{$last_transcript}}){

if ($qs < $min_qstart2){

$min_qstart2 = $qs;

}

}

print LOG "end_of_first_transcript: $max_qend1, start_of_last_transcript: $min_qstart2\n";

# deal with incompleteness

my $end_of_first_transcript;

# first transcript

# test direction of first transcript

if ($sstart{$first_transcript}{$max_qstart1} < $send{$first_transcript}{$max_qstart1}){

$end_of_first_transcript = $max_qend1 + ($len{$first_transcript} - $send{$first_transcript}{$max_qstart1});

}

else {

$end_of_first_transcript = $max_qend1 + ($send{$first_transcript}{$max_qstart1} - 1);

}

my $start_of_last_transcript;

# last transcript

# test direction of last transcript

if ($sstart{$last_transcript}{$min_qstart2} < $send{$last_transcript}{$min_qstart2}){

$start_of_last_transcript = $min_qstart2 - ($sstart{$last_transcript}{$min_qstart2} - 1);

}

else {

$start_of_last_transcript = $min_qstart2 - ($len{$last_transcript} - $sstart{$last_transcript}{$min_qstart2});

}

print LOG "corrected for incompleteness\nend_of_first_transcript: $end_of_first_transcript, start_of_last_transcript: $start_of_last_transcript\n";

my $dist = 0;

# test whether transcripts are overlapping

if ($end_of_first_transcript > $start_of_last_transcript){

$dist = int(($end_of_first_transcript - $start_of_last_transcript)/2);

$end_of_first_transcript -= $dist;

$start_of_last_transcript += $dist;

}

print LOG "overlap between transcripts: $dist\n";

print LOG "corrected for overlap\nend_of_first_transcript: $end_of_first_transcript, start_of_last_transcript: $start_of_last_transcript\n";

# overlap not too large

if ($dist < 150){

my $first_transcript_seq = substr($query,1,$end_of_first_transcript);

my $last_transcript_seq = substr($query,$start_of_last_transcript,$iso_length-$start_of_last_transcript+1);

# write transcripts

open (SPLIT, ">>$output_folder/split_chimers.txt");

my $gene_name;

my $isoform_number;

if ($isoform =~ /(TRINITY_DN\d+_c\d+_g\d+)_(i\d+)/){

$gene_name = $1;

$isoform_number = $2;

print SPLIT ">$gene_name" . "a_$isoform_number\n";

print SPLIT "$first_transcript_seq\n";

print SPLIT ">$gene_name" . "b_$isoform_number\n";

print SPLIT "$last_transcript_seq\n";

}

close(SPLIT);

print LOG "split $isoform into orthologs of $subject1 and $subject2\n\n";

$is_chimer = 1;

}

else {

print LOG "overlap too large for orthologs of $subject1 and $subject2 on $isoform\n\n";

}

}

else {

print LOG "only found BLAST hit for one ortholog\n";

}

close(LOG);

return $is_chimer;

}
