# Supplementary figures and images for: Comparative transcriptomics identifies candidate genes involved in the evolutionary transition from dehiscent to indehiscent fruits in Lepidium (Brassicaceae)
Source: BMC Plant Biol. 2022 Jul 14;22:340. doi: 10.1186/s12870-022-03631-8 (PMC9281134; doi:10.1186/s12870-022-03631-8)

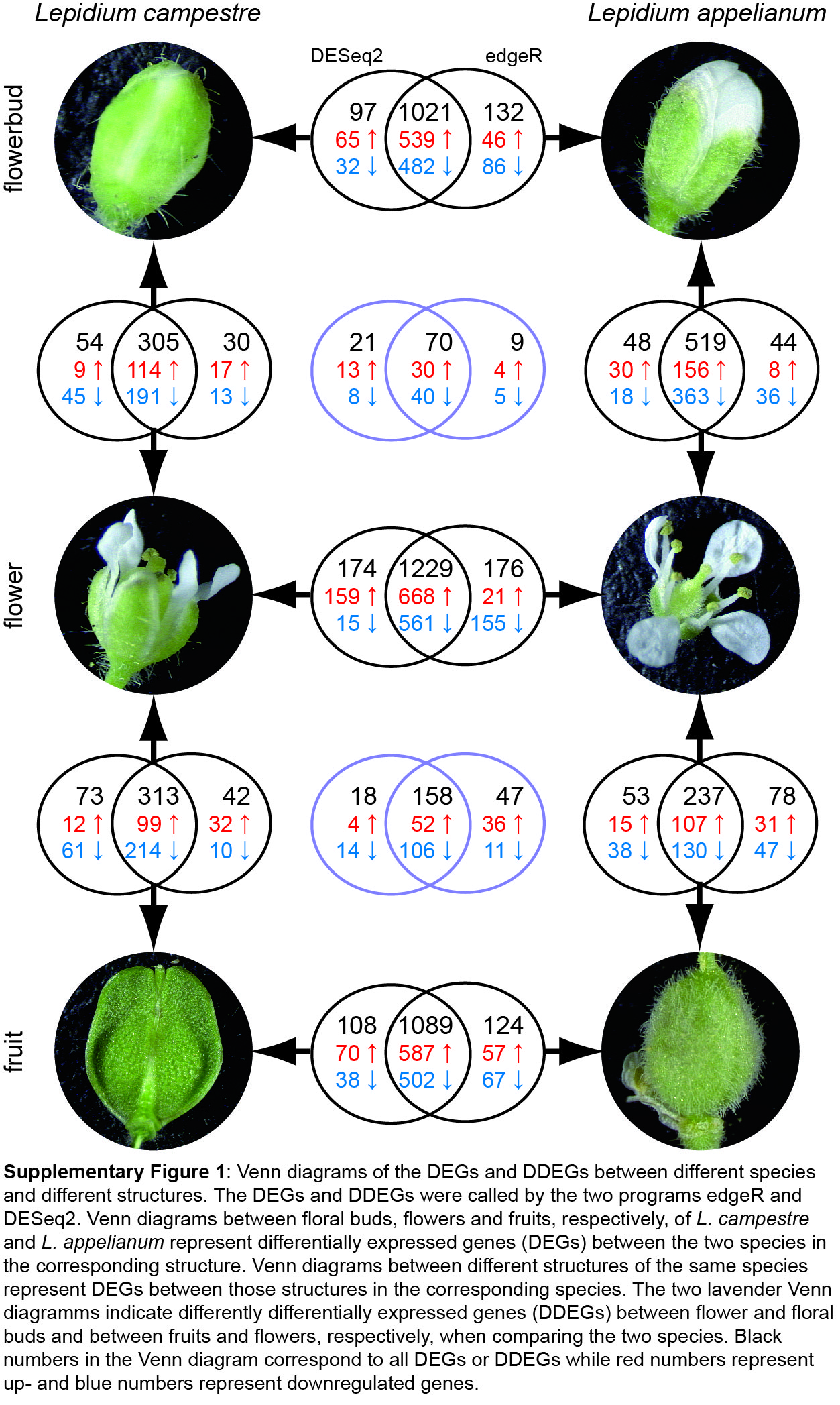

Supplement: Supplementary file 2 — Additional file 2: Supplementary Figure 1. [file 12870_2022_3631_MOESM2_ESM.jpg]

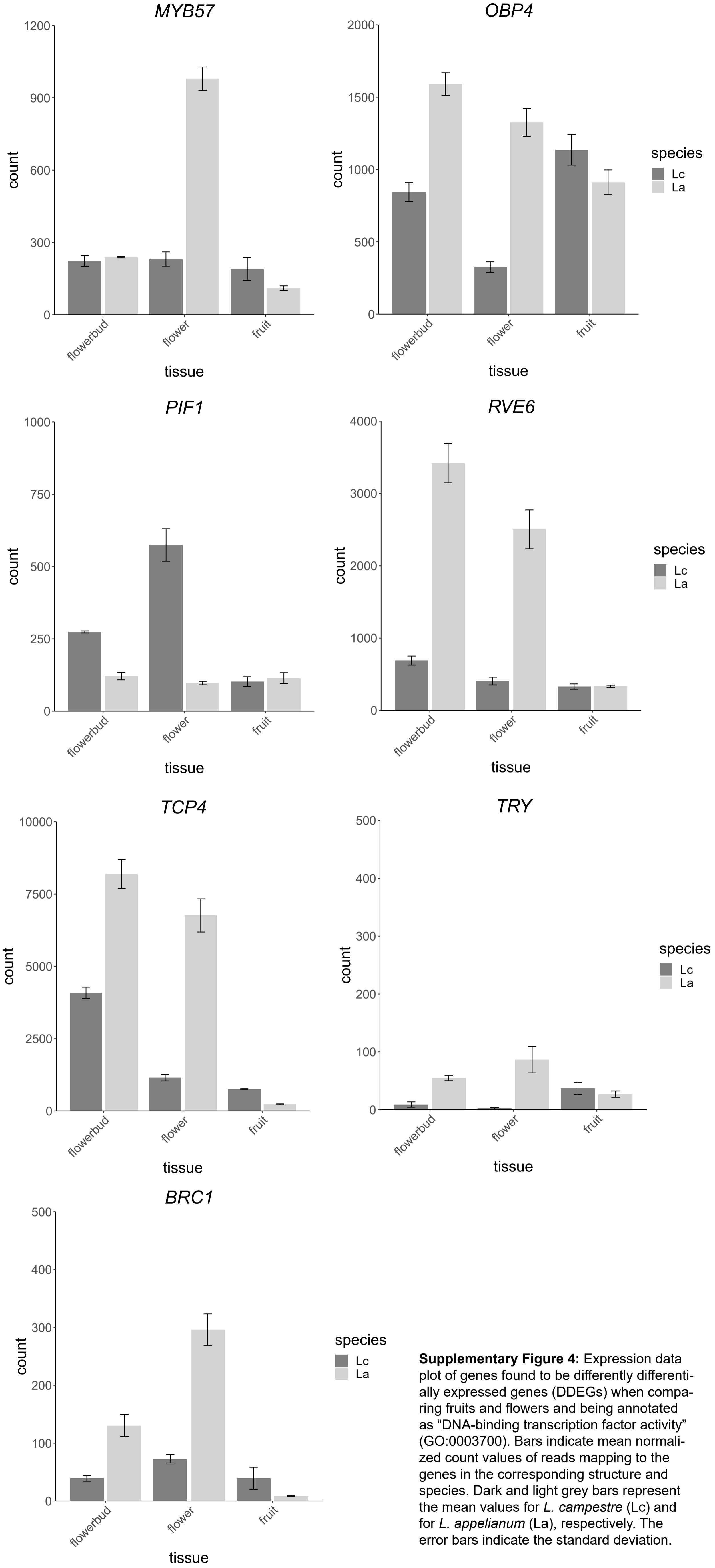

Supplement: Supplementary file 5 — Additional file 5: Supplementary Figure 4. [file 12870_2022_3631_MOESM5_ESM.pdf]
