# Supplementary material for: Comparative transcriptomics identifies candidate genes involved in the evolutionary transition from dehiscent to indehiscent fruits in Lepidium (Brassicaceae)
Source: BMC Plant Biol. 2022 Jul 14;22:340. doi: 10.1186/s12870-022-03631-8 (PMC9281134; doi:10.1186/s12870-022-03631-8)
Supplement: Supplementary file 3 — Additional file 3: Supplementary Figure 2. [file 12870_2022_3631_MOESM3_ESM.pdf]

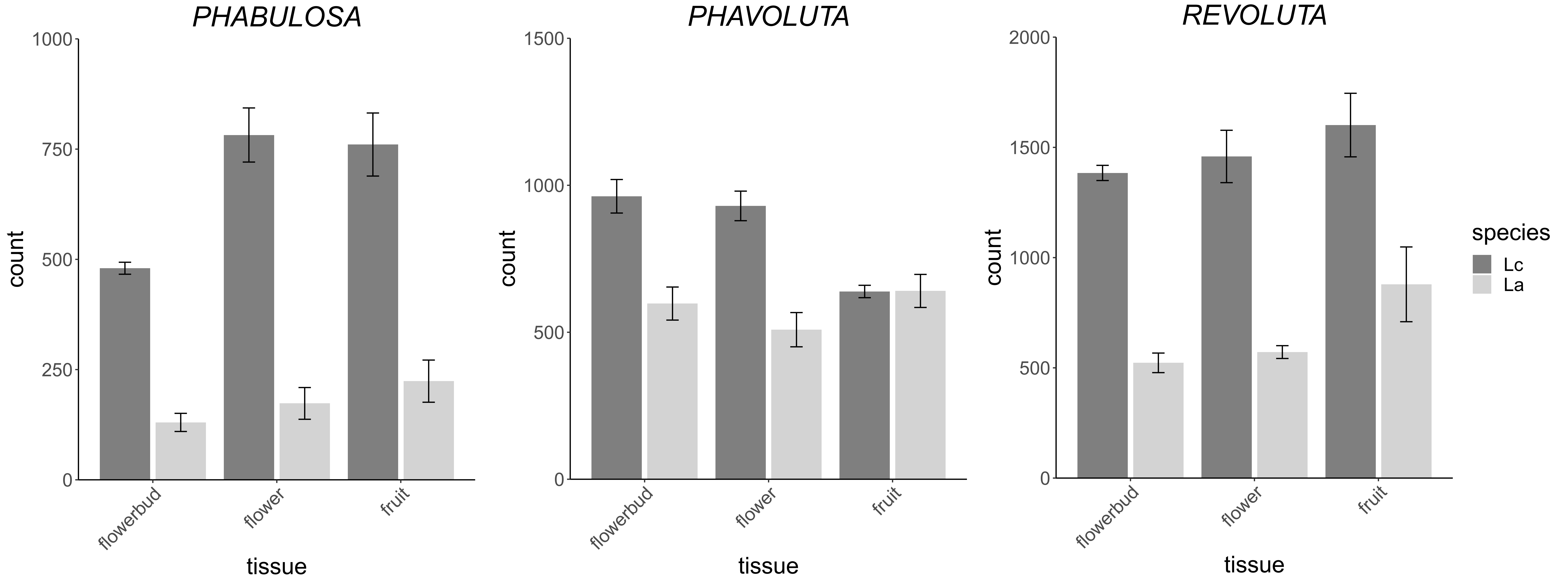

Supplementary Figure 2: Expression data plot of target genes to the miRNA homologous to miR165a-3p of *A. thaliana*. miR165a-3p is identical to miR165b, miR166a-3p, miR166b-3p, miR166c, miR166d, miR166e-3p, miR166f and miR166g such that they cannot be distinguished. Bars indicate mean normalized count values of reads mapping to the target genes in the corresponding structure and species. Dark and light grey bars represent the mean values for L. campestre (Lc) and for L. appelianum (La), respectively. The error bars indicate the standard deviation.
