# Supplementary material for: Comparative transcriptomics identifies candidate genes involved in the evolutionary transition from dehiscent to indehiscent fruits in Lepidium (Brassicaceae)
Source: BMC Plant Biol. 2022 Jul 14;22:340. doi: 10.1186/s12870-022-03631-8 (PMC9281134; doi:10.1186/s12870-022-03631-8)
Supplement: Supplementary file 4 — Additional file 4: Supplementary Figure 3. [file 12870_2022_3631_MOESM4_ESM.pdf]

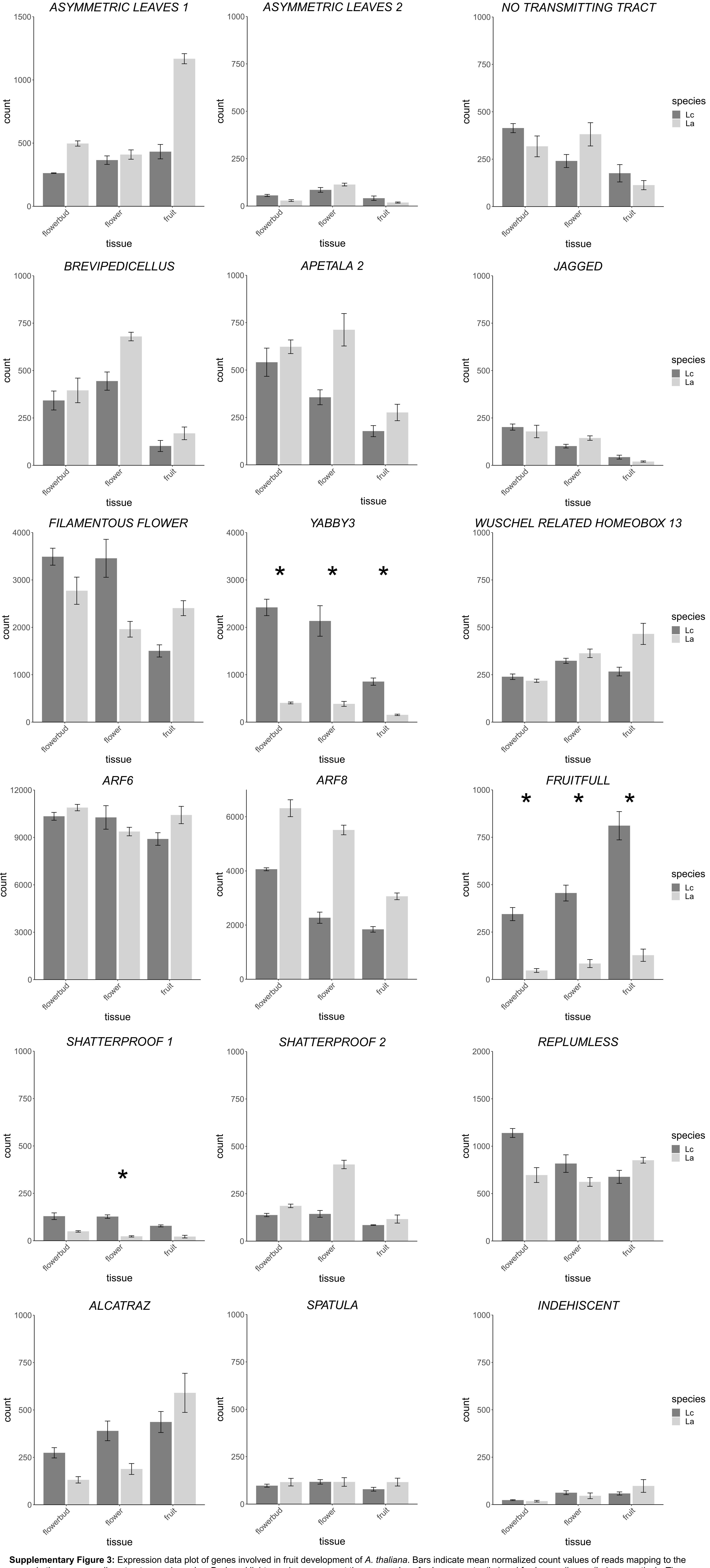

Supplementary Figure 3: Expression data plot of genes involved in fruit development of *A. thaliana*. Bars indicate mean normalized count values of reads mapping to the genes in the corresponding structure and species. Dark and light grey bars represent the mean values for *L. campestre* (Lc) and for *L. appelianum* (La), respectively. The error bars indicate the standard deviation. Significant differences between *L. appelianum* and *L. campestre* are indicated by asterisks ($P \leq 0.05$).
