# Supplementary material for: Comparative transcriptomics identifies candidate genes involved in the evolutionary transition from dehiscent to indehiscent fruits in Lepidium (Brassicaceae)
Source: BMC Plant Biol. 2022 Jul 14;22:340. doi: 10.1186/s12870-022-03631-8 (PMC9281134; doi:10.1186/s12870-022-03631-8)
Supplement: Supplementary file 6 — Additional file 6: Supplementary Table 1. Genes that were excluded from the ortholog transcriptome and which are annotated as “DNA-binding transcription factor activity” (GO:0003700). [file 12870_2022_3631_MOESM6_ESM.docx]

**Supplementary Table 1: Genes that were excluded from the ortholog transcriptome and which are annotated as “DNA-binding transcription factor activity” (GO:0003700).**

| *L. appelianum* | | | *L. campestre* | | | |  | |
| --- | --- | --- | --- | --- | --- | --- | --- | --- |
| Ortholog ID | Ortholog name | Ortholog description (based on TAIR) | | Ortholog ID | Ortholog name | Ortholog description (based on TAIR) | Reason for exclusion from ortholog transcriptome |  |
| AT1G02030.1 | n.a. | n.a. | | AT1G02030.1 | n.a. | n.a. | Length difference too large |  |
| AT1G02040.1 | n.a. | n.a. | | AT1G02040.1 | n.a. | n.a. | Length difference too large |  |
| AT1G08290.1 | WIP3 | n.a. | | AT1G08290.1 | WIP3 | n.a. | Alignment length too short |  |
| AT1G08320.1 | TGA9, bZIP21 | anther development | | AT1G08320.1 | TGA9, bZIP21 | anther development | No bidirectional BLAST hits |  |
| AT1G12260.1 | VND4, EMB2749, ANAC007, NAC007 | n.a. | | AT1G12260.1 | VND4, EMB2749, ANAC007, NAC007 | n.a. | Length difference too large |  |
| AT1G18570.1 | MYB51, AtMYB51, BW51A, BW51B, HIG1 | indolic glucosinolate biosynthesis | | AT1G18570.1 | MYB51, AtMYB51, BW51A, BW51B, HIG1 | indolic glucosinolate biosynthesis | Length difference too large |  |
| AT1G22810.1 | ATERF019, ERF019 | plant growth and senescence | | AT1G22810.1 | ATERF019, ERF019 | plant growth and senescence | Length difference too large |  |
| AT1G25330.1 | CES, CESTA, HAF, HALF FILLED | brassinosteroid biosynthesis | | AT1G25330.1 | CES, CESTA, HAF, HALF FILLED | brassinosteroid biosynthesis | Length difference too large |  |
| AT1G25550.1 | HHO3, HRS1 HOMOLOG3, NIGT1.1 | nitrate and phosphate signalling | | AT1G25550.1 | HHO3, HRS1 HOMOLOG3, NIGT1.1 | nitrate and phosphate signalling | Length difference too large |  |
| AT1G26590.1 | n.a. | n.a. | | AT1G26590.1 | n.a. | n.a. | No bidirectional BLAST hits |  |
| AT1G29010.1 | TIE3 | leaf development | | AT1G29010.1 | TIE3 | leaf development | No bidirectional BLAST hits |  |
| AT1G34650.1 | HDG10 | n.a. | | AT1G34650.1 | HDG10 | n.a. | Length difference too large |  |
| AT1G47655.1 | n.a. | n.a. | | AT1G47655.1 | n.a. | n.a. | Length difference too large |  |
| AT1G64625.1 | n.a. | male meiotic entry | | AT1G64625.1 | n.a. | male meiotic entry | Length difference too large |  |
| AT1G65910.1 | anac028, NAC028 | n.a. | | AT1G65910.1 | anac028, NAC028 | n.a. | Length difference too large |  |
| AT1G66230.1 | MYB20, AtMYB20 | secondary wall formation | | AT1G66230.1 | MYB20, AtMYB20 | secondary wall formation | Length difference too large |  |
| AT1G67260.1 | TCP1 | flower symmetry, brassinosteroid biosynthesis | | AT1G67260.1 | TCP1 | flower symmetry, brassinosteroid biosynthesis | Length difference too large |  |
| AT1G68360.1 | GIS3 | trichome initiation | | AT1G68360.1 | GIS3 | trichome initiation | Length difference too large |  |
| AT1G68640.1 | PAN | flower development, controlling AG expression | | AT1G68640.1 | PAN | flower development, controlling AG expression | No bidirectional BLAST hits |  |
| AT1G68800.1 | BRC2, TCP12 | Branching, similar but weaker than BRC1 | | AT1G68800.1 | BRC2, TCP12 | Branching, similar but weaker than BRC1 | Length difference too large |  |
| AT1G73870.1 | BBX16, COL7 | auxin biosynthesis | | AT1G73870.1 | BBX16, COL7 | auxin biosynthesis | Length difference too large |  |
| AT1G74480.1 | ATRKD2, RKD2, URP4 | female gametophyte development | | AT1G74480.1 | ATRKD2, RKD2, URP4 | female gametophyte development | Alignment length too short |  |
| AT1G79180.1 | ATMYB63, MYB63 | n.a. | | AT1G79180.1 | ATMYB63, MYB63 | n.a. | Length difference too large |  |
| AT1G80840.1 | WRKY40, ATWRKY40 | ABA response, pathogen response | | AT1G80840.1 | WRKY40, ATWRKY40 | ABA response, pathogen response | No bidirectional BLAST hits |  |
| AT2G01200.2 | IAA32 | n.a. | | AT2G01200.2 | IAA32 | n.a. | Length difference too large |  |
| AT2G01430.1 | ATHB17, ATHB-17, HB17 | stress tolerance | | AT2G01430.1 | ATHB17, ATHB-17, HB17 | stress tolerance | Length difference too large |  |
| AT2G16910.1 | AMS | tapetum and pollen development | | AT2G16910.1 | AMS | tapetum and pollen development | Length difference too large |  |
| AT2G23760.1 | BLH4, SAW2 | leaf serration, seed coat mucilage | | AT2G23760.1 | BLH4, SAW2 | leaf serration, seed coat mucilage | Length difference too large |  |
| AT2G31210.1 | BHLH091 | anther development | | AT2G31210.1 | BHLH091 | anther development | Length difference too large |  |
| AT2G33710.2 | n.a. | n.a. | | AT2G33710.2 | n.a. | n.a. | Alignment length too short |  |
| AT2G33810.1 | SPL3 | shoot development, floral transition | | AT2G33810.1 | SPL3 | shoot development, floral transition | Length difference too large |  |
| AT2G34830.1 | WRKY35, MEE24, AtWRKY35 | n.a. | | AT2G34830.1 | WRKY35, MEE24, AtWRKY35 | n.a. | Length difference too large |  |
| AT2G36890.1 | RAX2, MYB38, ATMYB38, BIT1 | shoot branching | | AT2G36890.1 | RAX2, MYB38, ATMYB38, BIT1 | shoot branching | No bidirectional BLAST hits |  |
| AT2G37260.1 | TTG2, ATWRKY44, WRKY44, DSL1 | trichome and root hair development | | AT2G37260.1 | TTG2, ATWRKY44, WRKY44, DSL1 | trichome and root hair development | Length difference too large |  |
| AT2G37740.1 | ATZFP10, ZFP10 | n.a. | | AT2G37740.1 | ATZFP10, ZFP10 | n.a. | Length difference too large |  |
| AT2G44745.1 | ATWRKY12, WRKY12 | pith development | | AT2G44745.1 | ATWRKY12, WRKY12 | pith development | Alignment length too short |  |
| AT2G45120.1 | ZAT4 | n.a. | | AT2G45120.1 | ZAT4 | n.a. | Length difference too large |  |
| AT3G01080.1 | WRKY58, ATWRKY58 | n.a. | | AT3G01080.1 | WRKY58, ATWRKY58 | n.a. | Length difference too large |  |
| AT3G04570.1 | AHL19 | Verticillium Wilt Resistance | | AT3G04570.1 | AHL19 | Verticillium Wilt Resistance | Length difference too large |  |
| AT3G10000.1 | EDA31 | Embryo sac development | | AT3G10000.1 | EDA31 | Embryo sac development | Length difference too large |  |
| AT3G13890.1 | MYB26, ATMYB26, MS35 | anther dehiscence | | AT3G13890.1 | MYB26, ATMYB26, MS35 | anther dehiscence | Alignment length too short |  |
| AT3G15170.1 | CUC1, ANAC054, ATNAC1 | carpel and ovule development, leaf development | | AT3G15170.1 | CUC1, ANAC054, ATNAC1 | carpel and ovule development, leaf development | No bidirectional BLAST hits |  |
| AT3G16500.1 | PAP1, IAA26 | regulation of Anthocyanin Biosynthesis | | AT3G16500.1 | PAP1, IAA26 | regulation of Anthocyanin Biosynthesis | Length difference too large |  |
| AT3G21330.1 | n.a. | n.a. | | AT3G21330.1 | n.a. | n.a. | Length difference too large |  |
| AT3G24500.1 | MBF1C, ATMBF1C | stress response | | AT3G24500.1 | MBF1C, ATMBF1C | stress response | Length difference too large |  |
| AT3G44290.1 | anac060, NAC060 | n.a. | | AT3G44290.1 | anac060, NAC060 | n.a. | Alignment length too short |  |
| AT3G45260.1 | BIB | root development | | AT3G45260.1 | BIB | root development | Length difference too large |  |
| AT3G46130.1 | ATMYB48, ATMYB48-3, MYB48 | n.a. | | AT3G46130.1 | ATMYB48, ATMYB48-3, MYB48 | n.a. | No bidirectional BLAST hits |  |
| AT3G48360.1 | BT2, ATBT2 | stress response | | AT3G48360.1 | BT2, ATBT2 | stress response | Length difference too large |  |
| AT3G51960.2 | ATBZIP24, BZIP24 | salt stress response | | AT3G51960.2 | ATBZIP24, BZIP24 | salt stress response | Alignment length too short |  |
| AT3G60580.1 | ZAT9 | n.a. | | AT3G60580.1 | ZAT9 | n.a. | Length difference too large |  |
| AT3G62610.1 | ATMYB11, PFG2, MYB11 | flavonol glycoside accumulation | | AT3G62610.1 | ATMYB11, PFG2, MYB11 | flavonol glycoside accumulation | Length difference too large |  |
| AT4G12240.1 | n.a. | n.a. | | AT4G12240.1 | n.a. | n.a. | Length difference too large |  |
| AT4G17600.1 | LIL3:1 | chlorophyll biosynthesis | | AT4G17600.1 | LIL3:1 | chlorophyll biosynthesis | Length difference too large |  |
| AT4G35280.1 | DAZ2 | Male Germ Line Development | | AT4G35280.1 | DAZ2 | Male Germ Line Development | Length difference too large |  |
| AT4G35390.1 | AGF1 | Gibberellin regulation | | AT4G35390.1 | AGF1 | Gibberellin regulation | Alignment length too short |  |
| AT4G35900.1 | FD, FD-1, atbzip14 | n.a. | | AT4G35900.1 | FD, FD-1, atbzip14 | n.a. | No bidirectional BLAST hits |  |
| AT5G01380.1 | n.a. | n.a. | | AT5G01380.1 | n.a. | n.a. | Length difference too large |  |
| AT5G06510.1 | NF-YA10 | disease development | | AT5G06510.1 | NF-YA10 | disease development | Length difference too large |  |
| AT5G07310.1 | ERF115 | root development | | AT5G07310.1 | ERF115 | root development | Alignment length too short |  |
| AT5G07500.1 | PEI1 | embryo development | | AT5G07500.1 | PEI1 | embryo development | Length difference too large |  |
| AT5G07580.1 | DEWAX2, ERF106 | cuticular wax biosynthesis | | AT5G07580.1 | DEWAX2, ERF106 | cuticular wax biosynthesis | Length difference too large |  |
| AT5G12870.1 | ATMYB46, MYB46 | secondary wall biosynthesis | | AT5G12870.1 | ATMYB46, MYB46 | secondary wall biosynthesis | Length difference too large |  |
| AT5G15830.1 | AtbZIP3, bZIP3 | leaf development | | AT5G15830.1 | AtbZIP3, bZIP3 | leaf development | Length difference too large |  |
| AT5G17260.1 | anac086, NAC086 | sieve element differentiation | | AT5G17260.1 | anac086, NAC086 | sieve element differentiation | Alignment length too short |  |
| AT5G22290.1 | anac089, NAC089 | stress response | | AT5G22290.1 | anac089, NAC089 | stress response | No bidirectional BLAST hits |  |
| AT5G45260.1 | RRS1, ATWRKY52, SLH1 | disease resistance | | AT5G45260.1 | RRS1, ATWRKY52, SLH1 | disease resistance | Alignment length too short |  |
| AT5G46590.1 | anac096, NAC096 | stress response | | AT5G46590.1 | anac096, NAC096 | stress response | Alignment length too short |  |
| AT5G52170.1 | HDG7 | n.a. | | AT5G52170.1 | HDG7 | n.a. | Length difference too large |  |
| AT5G61270.1 | PIF7 | red-light response | | AT5G61270.1 | PIF7 | red-light response | Length difference too large |  |
| AT5G62380.1 | VND6, ANAC101, NAC101 | xylem development | | AT5G62380.1 | VND6, ANAC101, NAC101 | xylem development | Length difference too large |  |
| AT5G65230.1 | AtMYB53, MYB53 | n.a. | | AT5G65230.1 | AtMYB53, MYB53 | n.a. | Length difference too large |  |
| AT5G66350.1 | SHI | plant growth | | AT5G66350.1 | SHI | plant growth | No bidirectional BLAST hits |  |
| AT1G02230.1 | ANAC004, NAC004 | n.a. | | AT1G02250.1 | anac005, NAC005 | vascular development |  |  |
| AT1G03790.1 | SOM | seed germination | | AT1G04240.1 | SHY2, IAA3 | root development |  |  |
| AT1G06180.1 | ATMYB13, ATMYBLFGN, MYB13 | n.a. | | AT1G06150.1 | EMB1444 | n.a. |  |  |
| AT1G12890.1 | n.a. | n.a. | | AT1G11490.1 | n.a. | n.a. |  |  |
| AT1G13300.1 | HRS1 | nitrate and phosphate signalling | | AT1G18835.1 | MIF3 | n.a. |  |  |
| AT1G13600.1 | AtbZIP58, bZIP58 | n.a. | | AT1G35490.1 | n.a. | n.a. |  |  |
| AT1G15580.1 | IAA5, ATAUX2-27, AUX2-27 | stress tolerance | | AT1G52880.1 | NAM, ANAC018, ATNAM, NARS2 | Embryogenesis |  |  |
| AT1G18400.1 | BEE1 | Transmitting Tract Development | | AT1G54330.1 | ANAC020, NAC020 | root development |  |  |
| AT1G18860.1 | WRKY61, ATWRKY61 | n.a. | | AT1G56160.1 | ATMYB72, MYB72 | disease resistance |  |  |
| AT1G25340.1 | MYB116, AtMYB116 | n.a. | | AT1G62975.1 | n.a. | n.a. |  |  |
| AT1G29860.1 | WRKY71, ATWRKY71 | shoot branching, flowering time | | AT1G63040.1 | n.a. | n.a. |  |  |
| AT1G31050.1 | PFA1 | n.a. | | AT1G68840.1 | RAV2, RAP2.8, TEM2, EDF2, AtRAV2 | floral induction |  |  |
| AT1G32240.1 | KAN2 | Repression of AS2 | | AT1G75250.1 | ATRL6, RSM3, RL6 | n.a. |  |  |
| AT1G32770.1 | ANAC012, SND1, NST3, NAC012 | secondary wall biosynthesis | | AT2G18300.3 | HBI1 |  |  |  |
| AT1G34670.1 | AtMYB93, MYB93 | lateral root development | | AT2G24300.2 | CBP60E | n.a. |  |  |
| AT1G43160.1 | RAP2.6 | abscisic acid signaling | | AT2G28160.1 | FIT1, ATBHLH029, FRU, BHLH029, ATBHLH29, ATFIT1 | iron uptake |  |  |
| AT1G44830.1 | ATERF014, EPI1, ERF014 | plant immunity | | AT2G29060.1 | n.a. | n.a. |  |  |
| AT1G54160.1 | NFYA5, NF-YA5 | drought resistance | | AT2G35550.1 | BPC7, BBR, ATBPC7 | n.a. |  |  |
| AT1G54330.1 | ANAC020, NAC020 | n.a. | | AT2G37000.1 | n.a. | circadian clock |  |  |
| AT1G63030.1 | ddf2 | n.a. | | AT2G39880.1 | MYB25, AtMYB25 | stress response |  |  |
| AT1G64000.1 | WRKY56, ATWRKY56 | n.a. | | AT2G40350.1 | n.a. | n.a. |  |  |
| AT1G64800.1 | n.a. | n.a. | | AT2G40970.1 | MYBC1 | n.a. |  |  |
| AT1G66470.1 | RHD6 | root hair development | | AT2G42660.1 | n.a. | n.a. |  |  |
| AT1G74660.1 | MIF1 | n.a. | | AT3G01140.1 | MYB106, NOK, AtMYB106 | trichome development |  |  |
| AT1G78080.1 | RAP2.4 | wound response, regeneration | | AT3G08500.1 | MYB83, AtMYB83 | cell wall biosynthesis |  |  |
| AT1G80730.1 | ZFP1, ATZFP1 | shoot development | | AT3G12250.4 | TGA6, BZIP45 | salicylic acid response |  |  |
| AT2G01500.1 | PFS2, WOX6, HOS9 | ovule patterning, repression of AG | | AT3G17100.1 | AIF3 | cell elongation |  |  |
| AT2G02540.1 | ATHB21, ZFHD4, HB21, ZHD3 | n.a. | | AT3G17730.1 | anac057, NAC057 | n.a. |  |  |
| AT2G04038.1 | AtbZIP48, bZIP48 | n.a. | | AT3G18650.1 | AGL103 | n.a. |  |  |
| AT2G17180.1 | DAZ1, ZAT2 | germ cell division | | AT3G19500.1 | n.a. | n.a. |  |  |
| AT2G20080.1 | n.a. | n.a. | | AT3G27920.1 | GL1, ATMYB0, ATGL1, MYB0 | trichome development |  |  |
| AT2G22750.2 | n.a. | n.a. | | AT3G29035.1 | ATNAC3, ANAC059, NAC3 | senescence |  |  |
| AT2G26150.1 | ATHSFA2, HSFA2 | heat stress | | AT3G56970.1 | BHLH038, ORG2 |  |  |  |
| AT2G27220.2 | BLH5 | n.a. | | AT4G02670.1 | AtIDD12, IDD12 | n.a. |  |  |
| AT2G31180.1 | ATMYB14, MYB14 | AT, MYB14 | | cold tolerance | AT4G11070.1 | WRKY41, AtWRKY41 |  |  |
| AT2G41690.1 | AT-HSFB3, HSFB3 | n.a. | | AT4G17785.1 | MYB39 | root development |  |  |
| AT2G43140.2 | BHLH129 | n.a. | | AT4G19520.1 | n.a. |  |  |  |
| AT2G44910.1 | ATHB4, ATHB-4, HB4 | organ polarity | | AT4G21340.1 | B70 | n.a. |  |  |
| AT2G45430.1 | AHL22 | hypocotyl development | | AT4G22680.1 | MYB85, AtMYB85 | lignin biosynthesis |  |  |
| AT2G46130.1 | WRKY43, ATWRKY43 | fatty acid accumulation | | AT4G23810.1 | WRKY53, ATWRKY53 | senescence |  |  |
| AT2G46990.1 | IAA20 | protoxylem development | | AT4G25490.1 | CBF1, DREB1B, ATCBF1 | stress tolerance |  |  |
| AT2G47070.1 | SPL1 | cu homeostasis | | AT4G32280.1 | IAA29 | hypocotyl growth |  |  |
| AT3G12720.1 | ATY53, ATMYB67, MYB67 | n.a. | | AT4G36740.1 | HB-5, ATHB40, HB40 | n.a. |  |  |
| AT3G18400.1 | anac058, NAC058 | repression of silique senescence | | AT4G38340.1 | n.a. | n.a. |  |  |
| AT3G21880.1 | BBX10, COL12 | regulation of flowering time and plant architecture | | AT5G01860.1 | n.a. | n.a. |  |  |
| AT3G22760.1 | SOL1 | stomata development | | AT5G03790.1 | ATHB51, LMI1, HB51 | leaf development, meristem identity |  |  |
| AT3G49690.1 | RAX3, MYB84, ATMYB84 | shoot branching | | AT5G10970.1 | n.a. | n.a. |  |  |
| AT3G49930.1 | n.a. | n.a. | | AT5G14010.1 | KNU | gynoecium development |  |  |
| AT3G49950.1 | n.a. | n.a. | | AT5G18560.1 | PUCHI | root development, flower initiation |  |  |
| AT3G52440.1 | n.a. | n.a. | | AT5G21960.1 | ERF016 | n.a. |  |  |
| AT3G53600.1 | ZAT18 | drought resistance | | AT5G25160.1 | ZFP3 | seed germination |  |  |
| AT3G60460.1 | DUO1 | Male Germ Line Development | | AT5G46830.1 | ATNIG1, NIG1 | stamen development and seed production |  |  |
| AT3G63350.1 | AT-HSFA7B, HSFA7B | salt stress response | | AT5G51790.1 | n.a. | n.a. |  |  |
| AT3G66656.1 | AGL91 | endosperm development | | AT5G52020.1 | n.a. | n.a. |  |  |
| AT4G01520.1 | anac067, NAC067 | n.a. | | AT5G53290.1 | CRF3 | root development |  |  |
| AT4G09820.1 | TT8, BHLH42 | proanthocyanidin biosynthesis, Fatty Acid Accumulation AT5G55020.1 | | AT5G55020.1 | ATMYB120, MYB120 | Pollen Tube Differentiation |  |  |
| AT4G12050.1 | AHL26 | n.a. | | AT5G56960.1 | n.a. | n.a. |  |  |
| AT4G14465.1 | AHL20 | regulation of plant immunity | | AT5G59340.1 | WOX2 | embryonic patterning |  |  |
| AT4G17490.1 | ATERF6, ERF-6-6, ERF6 | leaf growth and stress tolerance | | AT5G59820.1 | RHL41, ZAT12 | stress response |  |  |
| AT4G17710.1 | HDG4 | n.a. | | AT5G61620.1 | n.a. | n.a. |  |  |
| AT4G17800.1 | AHL23 | n.a. | |  |  |  |  |  |
| AT4G21440.1 | ATMYB102, ATM4, MYB102 | plant resistance | |  |  |  |  |  |
| AT4G22950.1 | AGL19 | flowering time determination | |  |  |  |  |  |
| AT4G25410.1 | n.a. | n.a. | |  |  |  |  |  |
| AT4G28110.1 | AtMYB41, MYB41 | cell wall biosynthesis | |  |  |  |  |  |
| AT4G29930.3 | n.a. | n.a. | |  |  |  |  |  |
| AT4G34410.1 | RRTF1 | redox homeostasis | |  |  |  |  |  |
| AT4G36060.1 | bHLH11 | iron homeostasis | |  |  |  |  |  |
| AT4G36240.1 | GATA7 | n.a. | |  |  |  |  |  |
| AT5G04150.1 | BHLH101 | iron homeostasis | |  |  |  |  |  |
| AT5G14750.1 | WER, ATMYB66, WER1, MYB66 | epidermal cell patterning | |  |  |  |  |  |
| AT5G16770.1 | AtMYB9, MYB9 | suberin deposition | |  |  |  |  |  |
| AT5G18450.1 | n.a. | n.a. | |  |  |  |  |  |
| AT5G22570.1 | WRKY38, ATWRKY38 | plant defense | |  |  |  |  |  |
| AT5G40350.1 | MYB24, AtMYB24 | stamen maturation | |  |  |  |  |  |
| AT5G43840.1 | AT-HSFA6A, HSFA6A | stress response | |  |  |  |  |  |
| AT5G44160.1 | NUC | flowering time determination | |  |  |  |  |  |
| AT5G46350.1 | WRKY8, ATWRKY8 | defense response | |  |  |  |  |  |
| AT5G47670.1 | NF-YB6, L1L | embryo development | |  |  |  |  |  |
| AT5G57390.1 | AIL5, CHO1, EMK | lateral organ development, flower development | |  |  |  |  |  |
| AT5G57420.1 | IAA33 | n.a. | |  |  |  |  |  |
| AT5G57620.1 | MYB36, AtMYB36 | root development | |  |  |  |  |  |
| AT5G61850.1 | LFY, LFY3 | transition to flowering | |  |  |  |  |  |
| AT5G62430.1 | CDF1 | transition to flowering | |  |  |  |  |  |
| AT5G64810.1 | WRKY51, ATWRKY51 | hormone signalling | |  |  |  |  |  |
